# Sleep and subjective happiness between the ages 40 and 59 in relation to presbyopia and dry eye

**DOI:** 10.1371/journal.pone.0250087

**Published:** 2021-04-23

**Authors:** Kazuno Negishi, Masahiko Ayaki, Motoko Kawashima, Kazuo Tsubota

**Affiliations:** 1 Department of Ophthalmology, Keio University School of Medicine, Tokyo, Japan; 2 Otake Clinic Moon View Eye Center, Kanagawa, Japan; 3 Tsubota Laboratory, Inc., Tokyo, Japan; Aston University School of Life and Health Sciences, UNITED KINGDOM

## Abstract

**Purpose:**

The aim of this study was to explore the status of quality of life between the ages 40–59 in relation to presbyopia and dry eye.

**Methods:**

Near add power and preferred contact lens power were examined in 219 participants at three clinics. 2000 participants completed a web-based survey on presbyopic symptoms, symptomatic dry eye, sleep quality, and subjective happiness.

**Results:**

Mean preferred corrected visual acuity was less than 20/20 in women (vs men, P<0.01) who were more often prescribed under-corrected contact lenses, whereas men preferred full correction. According to the annual progression rate of near add power in men (0.1468D/year), the estimated difference in presbyopia progression between men and women was 0.75 years in the right eye, and 1.69 years in the left eye, implying men might suffer presbyopia earlier than women due to higher myopic power of daily use contact lenses. The web-based survey revealed that men reported lower subjective happiness than women (P<0.001) and earlier onset of presbyopic symptoms by 1.1–1.7 years (P<0.05). Men received their first reading glasses 0.8 years earlier than women (P = 0.066). Multiple regression analysis demonstrated that awareness of presbyopic symptoms, visual burden, and dry eyes were significantly correlated with poor sleep quality and subjective happiness.

**Conclusion:**

Presbyopia and dry eye were significantly associated with sleep quality and subjective happiness in middle-adulthood.

## Introduction

Many middle-aged people may suffer presbyopia and dry eye. Presbyopia continuously progresses with aging and eventually requires most people to use near visual aids, in contrast to cataract and glaucoma, other common organic causes of loss of vision, that are treated by medical or surgical intervention [1–3]. Difficulty focusing is usually first experienced during the 30s to 40s depending on refraction and other visual factors in daily life and work. Most people initially adapt by increasing the distance from an object or removing myopic glasses. However, blurring, headache, eye pain, eye fatigue, neck pain, shoulder pain, and even productivity loss, often accompany difficulty focusing when it has become constant [4–8]. In the modern digital society, smartphone and computer displays with small letters are widely used across all generations, and the large impact of presbyopia on the economy and quality of life has emerged as a social problem in middle adulthood [9].

Dry eye disease (DED) is another prevalent geriatric eye disease, and may worsen with digital displays due to decreased blinking [10] and emitted blue light [11, 12]. DED is predominant in women, affecting quality of life (QOL), sleep, depression, and happiness [13–16]. QOL may worsen with presbyopia since difficulty focusing on near objects is a considerable burden in daily life. Recent survey results described a significant association between presbyopia and DED even after matching age/gender and comorbidity conditions [17] indicating these two disorders may concordantly worsen QOL. Middle-aged women more often work in professions in indoor settings that require near vision. Indeed, previous studies demonstrated women needed stronger near add power (the minimal additional power required to achieve sufficient near acuity under full distance refractive correction) than men [6–8]. Therefore, women may be more affected by presbyopia than men.

The deterioration of QOL due to ocular diseases could be measured with validated questionnaires. The Subjective Happiness Scale (SHS) [18, 19] has been used for DED, LASIK, and cataract surgery [20–22], and is closely associated with longevity [23]. The Pittsburg Sleep Quality Index (PSQI) is a sophisticated sleep quality scale [24, 25] assessing sleep disorders, which are strongly associated with life expectancy, cardiovascular disease, metabolic disorders, and hypertension [26–28]. However, to date, few studies described QOL in presbyopia [29].

The aim of this study was to explore the QOL in middle adulthood in relation to presbyopia and DED. Two complementary studies were carried out. The clinical study was conducted to determine the most recent preference of correction with contact lenses in middle adulthood in a visit to an vision care eye clinic. Near add power and preferred contact lens power for daily use were examined. The results of full ocular examinations by board-certified ophthalmologists and certified technicians would serve as a current clinical baseline data of presbyopia in middle adulthood. To further assess impact of presbyopia and DED on QOL, the web-based survey was performed to measure QOL-associated major indices including subjective happiness and sleep quality. Special attention was paid to sex differences in the onset and severity of presbyopia symptoms and burden.

## Materials and methods

### Clinical study

#### Study design and participants

This study was a clinic-based, retrospective, cross-sectional study involving healthy subjects attending the Jiyugaoka Ekimae Eye Clinic, Komoro Kosei General Hospital, and Tsukuba Central Hospital. The Institutional Review Board and Ethics Committee of the Tsukuba Central Hospital approved this study (approved on December 12, 2014, permission number 141201) and Jiyugaoka Ekimae Eye Clinic was approved as a collaborating institute of Tsukuba Central Hospital. Participants were recruited from April 2015 to March 2017 in these two institutions. The Institutional Review Board and Ethics Committee of Komoro Kosei General Hospital (approved on July 4, 2016, permission number 2802) approved this study and participants were recruited from July 2016 to March 2017. This study was carried out in accordance with the Declaration of Helsinki. The need for consent was waived by the Institutional Review Board. Consecutive patients were analyzed during the study period. Consecutive patients were analyzed during the study period.

This clinical study was a retrospective chart review since all examinations were routinely performed for contact lens prescription in participating clinics and hospitals. Every patient completed a health check sheet that included medical and family history before examination for contact lens prescription.

#### Inclusion and exclusion criteria

Participants aged 40 to 59 years visiting for contact lens prescription with bilateral phakic eyes and best-corrected visual acuity above 20/20 were included in the study. Individuals were excluded if they had glaucoma, vitreoretinal disease, any ocular surgery in the previous month, or acute ocular disease in the previous two weeks.

#### Ophthalmological examinations

Board-certified ophthalmologists examined all patients and excluded subjects with major age-related eye diseases, including cataract, glaucoma, and macular diseases. Ophthalmological evaluation of participants consisted of best-corrected visual acuity (Vision Chart, SSC-370^R^, Nidek Co., Ltd., Gamagori, Japan), autorefractometry (TonorefTM II, Nidek Co., Ltd., Aichi, Japan), slit-lamp biomicroscopy, funduscopy, and intraocular pressure measurements (TonorefTM II, Nidek Co., Ltd., Aichi, Japan). Binocular near add power was measured by a blinded examiner at a distance of 30 cm using a Bankoku near-acuity chart (Handaya Inc., Tokyo, Japan) or an automatic optometry system (AOS-700^R^; Nidek Co., Ltd., Gamagori, Japan) [30]. After determining the patient’s distance refractive correction, the minimal additional power required to achieve near acuity above 20/25 at 30 cm was measured in 0.25 D increments, and was recorded as near add power. Contact lens over-refraction was performed to determine participants preferred corrective power. Visual acuity for distance and near was measured using a decimal chart and converted to LogMAR for analysis. DED-related examinations consisted of tear break-up time, corneal staining test, and Schirmer test. They were carried out according to standard procedures [31–33]. The BUT was measured using wet fluorescein filter paper (Ayumi Pharmaceutical, Tokyo, Japan), applied at the lower lid margin. The fluorescein strip was wet with saline and the excess was flicked off. It was viewed with a suitable light source and yellow filter [34]. The BUT was defined as the time interval between the third blink and the appearance of the first dark spot in the cornea and was measured using a stopwatch. This was calculated with the mean of three measurements. A corneal staining score was determined to grade corneal epitheliopathy; graded at 0–2 for severity and area. Schirmer’s test was performed without topical anaesthesia. Strips of filter paper (Whatman No. 41; Showa Yakuhin Kako, Tokyo, Japan) were placed for 5 min at the outer third of the temporal lower conjunctival fornix, with the subject blinking spontaneously. The strips were then removed, and the length of the filter paper wetted by the spontaneous blinks was recorded (mm). Examination rooms were kept at 21–24 degrees centigrade and 40–60% humidity according to recommendation of Japanese Ministry of Health and Labor.

### Web-based survey

#### Ethical approval and participant recruitment

The web-based survey was approved by the Institutional Review Board and Ethics Committee of the Keio University (Approved on July 29, 2016, permission number 2015–0280) and was carried out in accordance with the Declaration of Helsinki. In charge of the survey was Ipsos Incorporated (Tokyo, Japan), a company that is certified in the protection of personal information, and launched a survey website to recruit 2,000 people adjusted for age and gender. All subjects aged 40 to 59 years who used the web survey panel (Research Panel Incorporated, Tokyo, Japan) were asked to participate in this study. Entry requirement to the survey was age and sex with potential presbyopia at the age of 40–59. Among 1,000,000 panels from the general public in Japan, age-matched participants were randomly selected, and invitation mails were sent without introducing the aim of the study. The first 2,000 participants who satisfied requirements were enrolled. The study took place from April 7 to April 13, 2017. Participants received no money, but reward points which could be used on the panel website as compensation.

Consent was waived by the ethics committee for this opt-out study. Instead, the first screen of the app stated that this was a research app and all the data obtained would be used for the study. It was also clearly stated that the participation was totally voluntary and could be withdrawn anytime, and their anonymity was preserved. Only after they pushed the agree button at the bottom of the screen, they could proceed to the next screen of the app. The participants were also provided with the contact information about the study and the inquiry form from the app.

#### Questionnaires

Participants were asked to complete two questionnaires on major indices for quality of life; happiness was evaluated with the validated Japanese version of SHS [19] and sleep quality was measured with a validated Japanese version of PSQI [25]. The SHS is a four-item questionnaire of subjective global happiness; each item requires patients to rate the statements on a 7-point Likert scale. The possible scores range from 1 to 7, and higher values corresponded to higher subjective happiness. The PSQI is comprised of seven subscales that evaluate sleep quality, including subjective sleep quality, daytime dysfunction, sleep latency, sleep duration, habitual sleep efficacy, sleep disturbances, and use of sleep medications; the score was calculated according to separate algorithms and analyzed. Each component was scored on a scale of 0 to 3, with 3 indicating the worst score. The highest possible global score is 21, and the normal range on the PSQI is less than 6. Questions on presbyopia included awareness and impact of difficulties focusing, age of first awareness of difficulty focusing, and age of first vision aid for near. The answers could include reading glasses, progressive spectacle lenses, monovision contact lenses, and multifocal contact lenses in reference to their uncorrected vision. (Tables 1 and 2). The questions on awareness discerned between a sensation of difficulty focusing without concerns and the sensation of difficulty focusing that affected daily life, henceforth referred to as awareness and impact. The severity of visual burden was asked for near, middle-distance, and far vision (1: No burden without blurred vision, 2: No burden with blurred vision, 3 Burden with blurred vision). A short dry eye questionnaire [35] was used to detect symptomatic dry eye with three questions that are widely used in epidemiological studies. The content of the short questionnaire included three questions: (1) How often do your eyes feel dry (not wet enough)?, (2) How often do your eyes feel irritated?, and (3) Have you ever been diagnosed (by a clinician) as having dry eye syndrome?

**Table 1:** Survey results of quality of life indices and ocular symptoms.

	Men (N = 1006)	Women (N = 994)	P-value*
Age	49.0 ± 5.7	49.0 ± 5.7	0.891
SHS	4.16 ±1.0	4.38 ± 1.1	< 0.001*
PSQI	5.46 ± 2.92	5.32 ± 2.88	0.293
Symptomatic DED (%)	28.4	42.7	< 0.001*
Awareness of difficulty focusing (%)	71.6	69.8	0.376
Impact of difficulty focusing (%)	47.5	46.6	0.162
Near correction (%)	26.3	26.6	0.879
Burden for near vision^A^	1.59 ± 0.85	1.51 ± 0.79	0.027*
Burden for middle-distance vision^A^	1.41 ± 0.72	1.40 ± 0.71	0.704
Burden for far vision^A^	1.47 ± 0.77	1.50 ± 0.71	0.445

*P<0.05, chi-squared test or *t*-test as appropriate.

^A^1 = No burden without blurred vision; 2 = No burden with blurred vision; 3 = Burden with blurred vision. Abbreviations; SHS, Subjective Happiness Scale, PSQI, Pittsburg Sleep Quality Index global score.

**Table 2:** Age of first awareness of difficulty focusing and first reading glasses.

Symptom and mean age (y)	Men	Women	⊿	P-value*
Hard to see small letters up close	43.9± 7.4 (6–59)	45.6± 7.0 (7–59)	1.7	< 0.001*
Hard to focus on small object up close	44.1± 7.7 (6–58)	45.5±6.6 (10–57)	1.4	0.010*
Hard to focus when changing focus from distance to near and near to distance	43.8±7.1 (10–56)	45.1 ±6.9 (14–57)	1.3	0.040*
See better when I increase distance from the object	45.6±4.9 (23–58)	46.7±4.8 (14–58)	1.1	0.021*
First reading glasses	47.4±5.2 (30–58)	48.2±4.4 (30–57)	0.8	0.066

*P<0.05, *t*-test. min and max values in parenthesis.

#### Statistical analysis

The sample size was calculated with a 0.05 margin of error, 95% confidence interval. Effect size and standard deviation were derived from a previous clinical study. For example, an effect size of 0.16D was identified in a previous study of accommodation [36]. Thus, an appropriate sample size for [preferred corrective power of contact lens–full correction power for far OD] was 5, calculated with a 0.05 margin of error, 95% confidence interval, effect size of 0.16, and standard deviation 0.088. Regarding the web-based study, an effect size of 0.68 in SHS was identified in a previous study of SHS [22]. An appropriate sample size for SHS was 950, calculated with a 0.05 margin of error, 95% confidence interval, an effect size of 0.68, and standard deviation 1.1.

Where appropriate, data are given as mean ± standard deviation since the obtained data were normally distributed using the Kolmogorov-Smirnov test. The visual acuity was converted to the logMAR. The difference of optical parameters and responses between men and women were analyzed using unpaired *t*-test and chi-squared test as appropriate. Correlations were evaluated using a standardized partial regression coefficient. Regression analysis was performed to identify the factors affecting SHS and PSQI by simple correlation. Multiple regression analysis was then performed to determine the predictors of SHS and PSQI, and possible predictors (responses and presence) of DED. All analyses were performed using StatFlex (Atech, Osaka, Japan) with p < 0.05 considered significant.

## Results

### Clinical study

**Fig 1:**
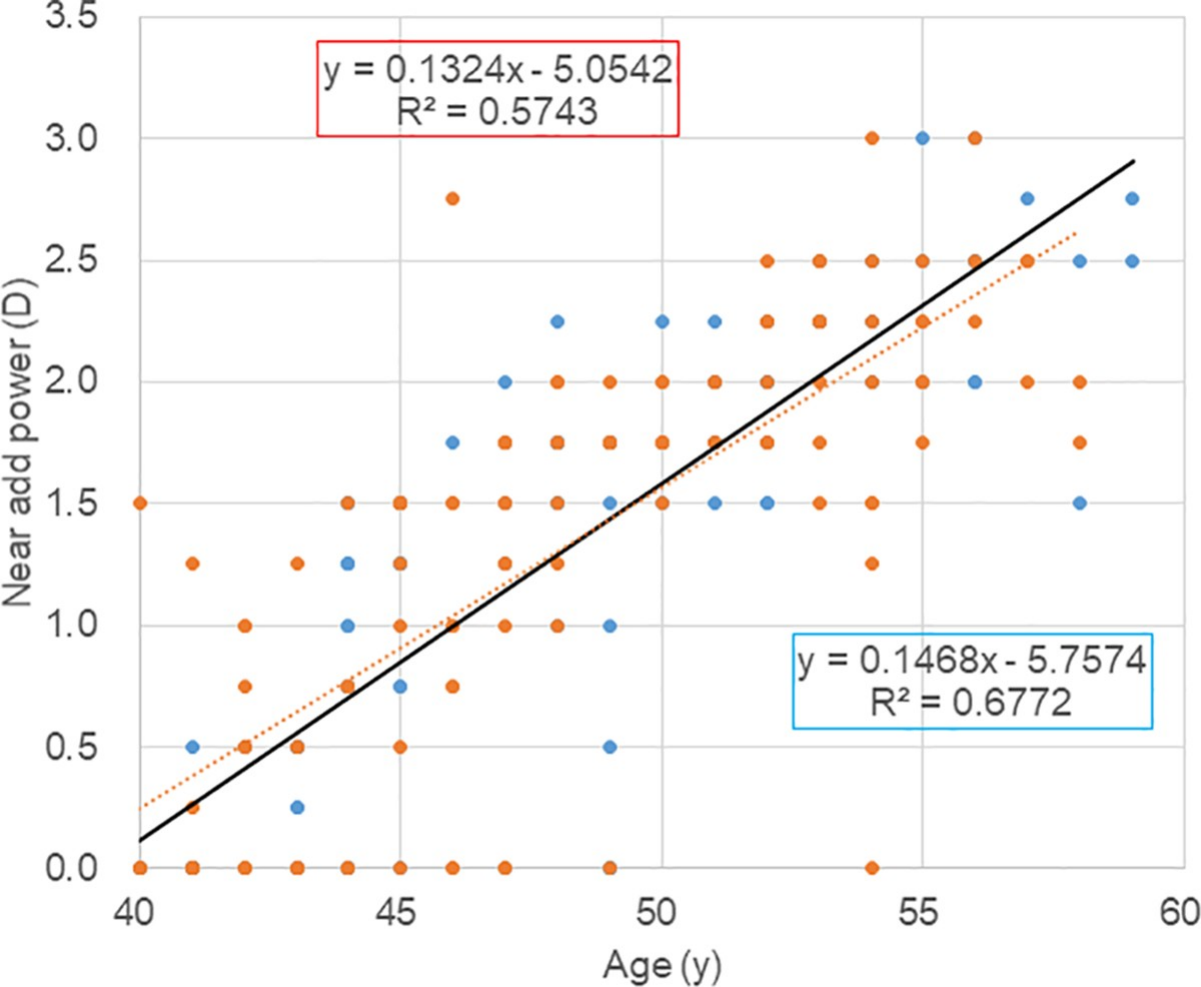
Scatter plots of near add power and age. Annual progression of near add power was similar in men (black circle, solid regression line) and women (red triangle, dotted regression line). Symbol overlap may not accurately represent the number of participants. The number of zero near power cases was 21 men and 26 women with no difference. It is also indicated by similar regression lines of both groups.

One hundred and six male and 113 female age-matched participants were enrolled in the clinical study. Refractive parameters were similar in both sexes (Table 3 and S1 Data). The clinical study showed that the distribution of refraction was high myopia (≤ -6.00D)(33.0%), mild myopia (> -6.00D)(61.3%), and hyperopia (5.7%) in men and high myopia (36.3%), mild myopia (61.9%), and hyperopia (1.8%) in women. During the examination for prescription of contact lenses for daily use, many women did not prefer best corrected power for their contact lens, and were prescribed with under-corrected power compared with men (men vs women, *P* = 0.003 in the right eye and *P* = 0.007 in the left eye). Scatter plots of near add power indicated a similar slope of the regression line in both sexes (Fig 1). There were 47 patients with zero diopter add power and all were myopic. Mean age was 43.3±2.9 (40–49) in men (n = 21) and 43.4±3.3 (40–54) in women (n = 26), respectively (*P* = 0.922, *t*-test). The spherical equivalent in the right eye was −4.81±2.10D (−1.75-−8.50D) in men and −4.46±2.15D (−0.12-−8.25D) in women, respectively (*P* = 0.580, *t*-test). All DED-related parameters were predominant in women.

**Table 3:** Refraction, near add power, and dry eye-related parameters in men and women.

	Men (N = 106)	Women (N = 113)	P-value*
Age	48.1 ±5.1	48.1±4.9	0.828
Near add power (D)	1.28±0.89	1.30±0.86	0.841
Spherical equivalent OD (D)	−4.73 ±3.28	−4.48±3.05	0.536
Spherical equivalent OS (D)	−4.48±3.05	−4.81 ±2.71	0.397
Astigmatism OD (D)	0.48±0.52	0.55±0.62	0.378
Astigmatism OS (D)	0.52±0.61	0.54 ±0.62	0.827
Preferred corrective power of contact lens–full correction power for far OD (D)	0.37 ±0.58 (+2.75 - −0.75)	0.48 ± 0.50 (+2.50 - −0.5)	0.192
Preferred corrective power of contact lens–full correction power for far OS (D)	0.26 ±0.51 (+1.25 - −0.75)	0.51 ±0.57 (+3.00 - −1.00)	0.002*
Preferred visual acuity with contact lens OD	−0.007±0.071	−0.041 ±0.088	0.003*
Preferred visual acuity with contact lens OS	−0.008 ±0.077	−0.038±0.082	0.007*
DED-related parameters			
Tear break-up time (%, ≤5s)	31.2	55.4	< 0.001*
Corneal staining score	0.136±0.42	0.292±0.54	0.019*
Schirmer test (%, ≤5mm)	0	16.7	<0.001*
Dry eye medication (%)	28.9	75.3	< 0.001*

*P<0.05, chi-squared test or *t*-test as appropriate. DED, dry eye disease.

### Web-based study

**Fig 2:**
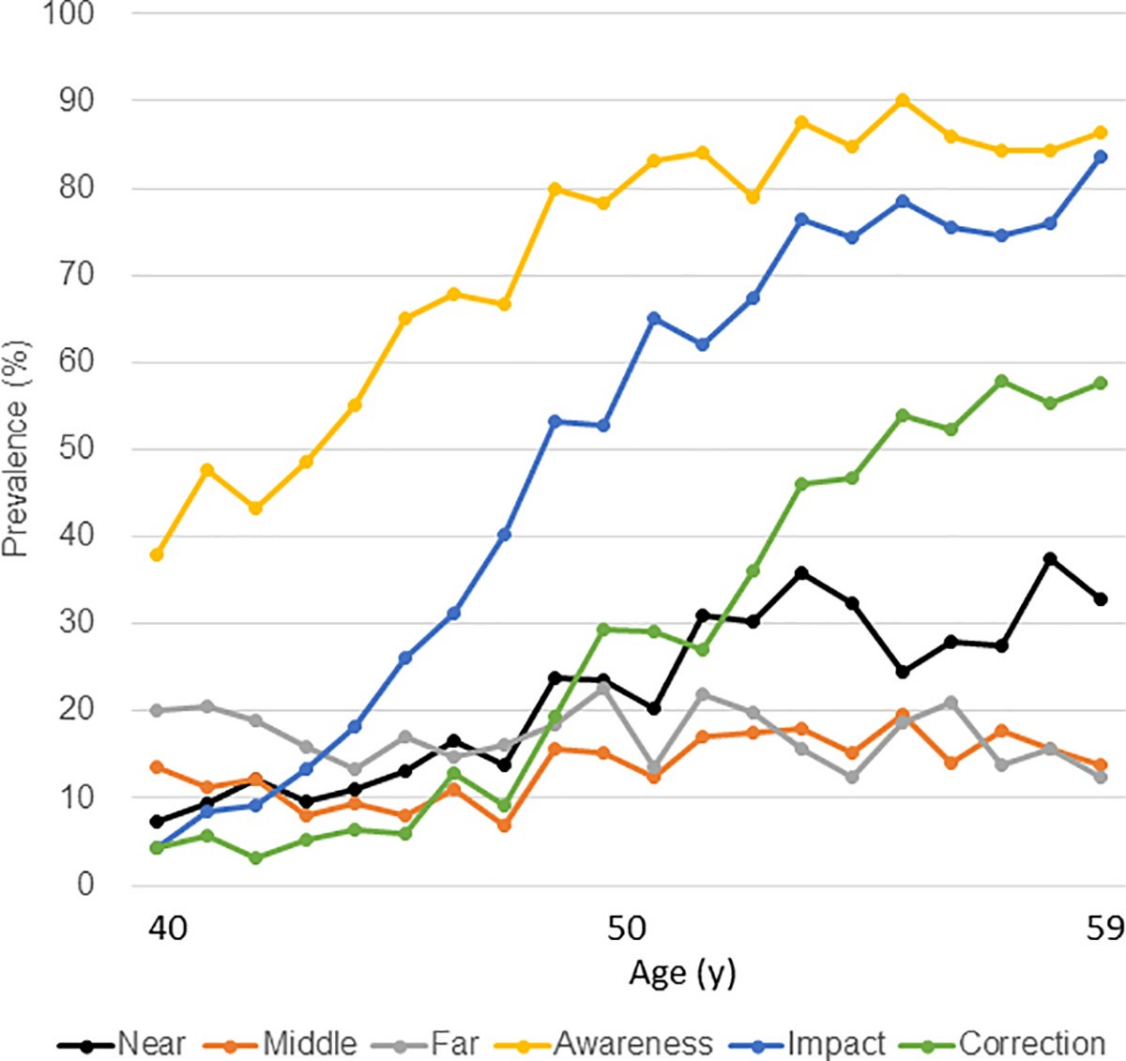
Prevalence of symptoms and burden with vision, and the proportion of near correction. The proportion of awareness of presbyopia was greater than that of impact across all ages surveyed. Awareness of difficulty focusing was age-dependent. The proportion of near correction was very low until age of 47 and then increased steeply. The severity of burden with near vision was apparently age-dependent, and burden with middle-distance vision were constant until increasing after age of 48, whilst burden with far vision was constant across all ages surveyed. Symbols and abbreviations: Near (black), burden with near vision; Middle (red), burden with middle-distance vision; Far (grey), burden with far vision; Awareness (orange), awareness of difficulty focusing; Impact (blue), reported impact of difficulty focusing; Correction (green), near correction.

The web-based survey revealed that the prevalence of awareness of difficulty focusing and the proportion of near correction was similar in both sexes (Table 1 and S2 Data). In contrast, burden with near vision was more serious in men (1.59±0.85) than women (1.51±0.79, *P* = 0.027). SHS was worse in men (4.16±1.00) than women (4.38±1.10; *P*< 0.001) and PSQI (5.46±2.92 for men and 5.32±2.88 for women) was similar (*P* = 0.293). Graphic presentation of the prevalence of symptoms and visual burden demonstrated that approximately 38% of participants already noticed difficulty focusing by the age of 40 years (Fig 2). The proportion of awareness of presbyopia was greater than that of impact across all ages surveyed. Despite an age-dependent increase of awareness of difficulty focusing, the proportion of near correction was very low until age 47, and then increased steeply. The severity of burden with near vision was apparently age-dependent, and burden with middle-distance vision were constant until slowing after age 48, whilst burden with far vision were constant across all ages surveyed.

The prevalence of symptomatic DED was much greater in women than men (P<0.001). The onset of men’s near vision symptoms was earlier by 1.1–1.7 years (P<0.05; Table 2). The mean age of men’s first reading glasses was 0.8 years earlier than in women (P = 0.066, 47.4 years vs 48.2 years). Eleven men and 7 women had their first reading glasses before age 40. Eight (44.4%) of them were hyperopic, whereas 13.7% of all participants were hyperopic. The duration from earliest awareness of symptoms to first reading glasses was 2.6 years in men and 3.1 years in women.

Simple regression analysis of SHS and PSQI revealed male sex, visual burden, and no near correction was correlated with low SHS, whereas presbyopic symptoms and visual burden were correlated with poor PSQI (Tables 4 and 5). Although the results of DED and presbyopia were comparable, the presence of symptomatic DED was more strongly correlated with SHS and PSQI than presbyopic parameters. Multiple regression analysis with visual symptoms demonstrated male sex was the strongest factor for low SHS (P<0.001) and male sex was also the strongest factor in the multiple regression analysis with visual burden for low SHS (P<0.001). Regarding PSQI, multiple regression analysis with visual symptoms demonstrated awareness of difficulty focusing was only significantly correlated with poor PSQI (P<0.001) after adjustment for age and sex, and multiple regression analysis with visual burden demonstrated significant correlation with poor PSQI (P<0.001).

**Table 4:** Regression analysis for happiness and sleep.

	^A^Happiness		^B^Sleep	
Parameters for simple regression	beta	P*	beta	P*
Age (y)	0.069	0.002	0.020	0.371
^C^Sex	-0.104	<0.001	0.023	0.293
Awareness of difficulty focusing	-0.025	0.272	0.140	<0.001
Impact of difficulty focusing	0.008	0.694	0.056	0.012
Near correction	0.052	0.020	0.032	0.156
Burden with near vision	-0.066	0.003	0.115	<0.001
Burden with middle distance vision	-0.046	0.038	0.063	0.005
Burden with far vision	-0.079	<0.001	0.101	<0.001
Dry eye	-0.087	<0.001	0.163	<0.001
Happiness	-	-	-0.384	<0.001
Sleep	-0.384	<0.001	-	-
Multiple regression, Model 1: Symptoms with focusing				
Age	0.082	0.002	-0.028	0.285
Sex	-0.102	<0.001	0.020	0.355
Awareness of difficulty focusing	-0.050	0.048	0.151	<0.001
Impact of difficulty focusing	-0.039	0.200	0.002	0.939
Near correction	0.053	0.052	-0.006	0.809
Multiple regression, Model 2: Burden with focusing				
Age	0.079	<0.001	0.002	0.925
Sex	-0.100	<0.001	0.017	0.422
Burden with near vision	-0.068	0.010	0.110	<0.001
Burden with far vision	-0.073	0.006	0.095	<0.001

*P<0.05, Standardized partial regression coefficient. A: Subjective happiness scale (0 (unhappy)-7 (happy)), B: Pittsburg Sleep Quality Index global score (higher score refers to poor sleep), C: Men = 1, Women = 0.

**Table 5:** Regression analysis for happiness and sleep stratified with sex.

	^A^Happiness				^B^Sleep			
	Men		Women		Men		Women	
Parameters	beta	P*	beta	P*	beta	P*	beta	P*
Age (y)	−0.042	0.178	−0.093	0.003*	0.040	0.200	0.000	0.982
Awareness of difficulty focusing	−0.071	0.032*	−0.035	0.295	0.166	< 0.001*	0.134	< 0.001*
Impact of difficulty focusing	−0.037	0.310	−0.038	0.318	0.074	0.044*	0.052	0.172
Near correction	0.075	0.031*	−0.025	0.490	0.005	0.878	0.053	0.135
Burden with near vision	−0.070	0.032*	−0.051	0.119	0.148	< 0.001*	0.109	0.001*
Burden with far vision	−0.085	0.007*	−0.065	0.039*	0.154	< 0.001*	0.081	0.011*
Dry eye	−0.133	< 0.001*	−0.074	0.019*	0.163	< 0.001*	0.177	< 0.001*
Happiness	–	–	–	–	−0.364	< 0.001*	−0.404	< 0.001*
Sleep	−0.364	< 0.001*	−0.404	< 0.001*	–	–	–	–
Multiple regression, Model 1: Symptom with focusing and DED								
Awareness of difficulty focusing	−0.058	0.103	−0.020	0.588	0.144	<0.001*	0.123	<0.001*
Impact of difficulty focusing	−0.058	0.160	−0.018	0.672	0.030	0.446	−0.031	0.482
Near correction	0.103	0.007	−0.011	0.783	−0.029	0.428	0.033	0.383
DED	−0.122	<0.001*	−0.071	0.024*	0.146	<0.001*	0.169	<0.001*
Model 2: Burden with vision								
Burden with near vision	−0.048	0.159	−0.039	0.244	0.111	0.001*	0.096	0.004*
Burden with far vision	−0.071	0.032*	−0.058	0.075	0.121	<0.001*	0.062	0.055

*P<0.05, Standardized partial regression coefficient.

A: Subjective happiness scale (0 (unhappy)-7 (happy))

B: Pittsburg Sleep Quality Index global score (higher score refers to poor sleep)

## Discussion

The clinical results suggest men suffer presbyopia more severely than women, which it is contrary to our initial hypothesis that women may be more affected by presbyopia than men. This apparent discrepancy is explained as follows. The clinical results also demonstrated women actually adapted to presbyopia by using under-corrected contact lenses. They suffer less severely from presbyopia although presbyopia affects both genders equally because the amplitude of accommodation is the same in men and women of the same age. These clinical results may indicate women tolerated near vision symptoms longer than men by using under-corrected optical devices for daily use. Our speculation is supported by a previous study [8] reporting a weak association between accommodative amplitude and gender, and differences in the addition requirements for near vision were due to different preferred viewing distances or uncorrected hyperopia. Beginning in adolescence, girls spend more time on smartphones and suffer less eye fatigue than boys [37, 38]. Clinical data and survey results both suggested men suffered presbyopia earlier and more severely than women. Our speculation is based on burden for near vison (Table 1), happiness (Table 5), and sleep (Table 5), however, the detected differences were small and the clinical relevance may be limited. Further study is warranted to confirm our results.

The results of regression analysis for web-based survey data indicated presbyopic symptoms and burden with near vision were significantly associated with subjective happiness and sleep quality. The present results may be deemed contrary to our initial hypothesis that women may be more affected by presbyopia than men. This apparent discrepancy is explained as follows. The clinical results also demonstrated women actually adapted to presbyopia by using under-corrected contact lenses. They suffer less severely from presbyopia although presbyopia affects both genders because equally the amplitude of accommodation is the same in men and women of the same age.

One previous study assessed QOL associated with presbyopia using utility and described a small decrease in QOL (utility = 0.980). 10% of participants had a presbyopia-associated utility of 0.95 or less [29]. They used preference-based time trade-off utility analysis to evaluate individual values, whereas the outcome measure of the current study was to quantitively measure emotion (subjective happiness) and health index (sleep quality). There is a substantial difference between the two studies, possibly because participants may have been sensitive to the indices used in the current study. There are some cases in zero near add power in women independently of the subject’s age. The discrepancy between add power and age could be clarified in future research, including retrospective or prospective longitudinal studies. Pupillary diameter, corneal multifocality, and aberration may contribute to this apparently enhanced accommodation [39–42]. DED is a very common disease in middle adulthood, being associated with decreased QOL, subjective happiness, and sleep quality as reported in numerous studies. The present results demonstrated DED was associated with happiness and sleep more strongly than presbyopia. It may be advisable that middle aged persons may suffer worsened QOL, even without vision-threatening eye disease. The web-based survey results suggested presbyopia may reasonably worsen QOL of many people between the ages 40–59 and, additionally, other ocular disorders should be considered to evaluate QOL in this age group. Detailed interview and examination of other ocular diseases and systemic comorbidity would be contributory to disclose QOL of middle adulthood.

The web-based survey results indicate that age 48 was a critical point for presbyopia when the majority of people first wear reading glasses. Near add power may plateau around 45–48 years of age [5] and patients may likely then first visit an optician to check their vision and then see a physician. Vision care personnel are recommended to advise on potential risks of deteriorated QOL for middle-aged men, preferring full correction. In conclusion, presbyopia and dry eye disease were significantly associated with sleep quality and subjective happiness in middle-adulthood.

The strength of the present clinical study and web-based survey was age- and sex-matching in a sufficiently large sample size, and recruitment of middle-aged participants covering the age from the initiation to the plateau phase of presbyopia. Monofocal contact lens power was chosen to explore preferred daily vision because most patients use them to see comfortably without spectacles. Thus, preferred power of daily monofocal contact lenses may cover typical focusing tasks on participants, and is a relevant indicator for evaluating the burden of presbyopia.

The current study has several limitations. First, pupil size was not measured and it is a significant methodological limitation. Second, the clinical study participants were comprised of contact lens users, i.e., mostly myopes due to availability of patients. Myopic subjects may suffer presbyopia more severely than hyperopes since they can see near without correction for many years. Therefore, further studies should involve hyperopes and emmetropes. The type of lens was not registered, which presents a limitation since the type of lens can affect dryness symptoms. Third, this was a clinic-based study and may not represent the general population. A community-based study would be necessary to generalize the results. Fourth, the impact of presbyopia depends on lifestyle, hobbies, and work, therefore a more detailed survey would reveal the interaction between presbyopia and QOL in the middle-aged population. Fifth, the current study was comprised of two different studies. A single study with one cohort would further confirm the results, although the combination of two studies effectively conveyed reasonable results. Finally, the statistical results obtained with the current study were nominal and clinical relevance may not be significant. Further study should be warranted to further confirm our results.

## Supporting information

S1 Data The raw data of the subjects participating clinical study. (XLSX)

S2 Data The raw data of the subjects participating web-based survey. (XLSX)
